# Digital Reminiscence Therapy for Caregivers of Family Members With Dementia

**DOI:** 10.1001/jamanetworkopen.2026.8286

**Published:** 2026-04-01

**Authors:** Leah E. Walsh, Emily Samuels, Robert M. Arnold

**Affiliations:** Brookdale Department of Geriatrics and Palliative Medicine, Icahn School of Medicine at Mount Sinai, New York, New York

Family and friend caregivers of individuals with Alzheimer disease or related dementias (ADRD) experience repeated, significant losses throughout the individual’s disease trajectory. As individuals with ADRD experience cognitive decline and behavioral and mood changes, caregivers witness firsthand small losses of their care partner that grow to represent the first death of the person for whom they care. Both the individual with ADRD and the caregiver have their previous relationship and previous sense of self stolen from them as part of the ADRD trajectory. This type of grief, termed *predeath grief*, begins long before bereavement and is characterized by longing for aspects of their care partner lost due to cognitive decline, or by role and identity confusion, given the transition from adult child or spouse to caregiver.^1^ Predeath grief is associated with worse bereavement outcomes,^2^ such as prolonged grief disorder, underscoring the need for intervention while caregivers are engaged in their role. An intervention that helps caregivers reconnect with meaningful aspects of their care partner, their own identity, and their relationship with their care partner may serve to buoy caregivers throughout the ADRD decline and into bereavement.

Falzarano et al^3^ found initial support for the feasibility, acceptability, and preliminary effects of the Living Memory Home for Dementia Care Pairs (LMH-4-DCP) intervention on predeath grief in a sample of family members caring for an individual with ADRD. LMH-4-DCP is a digital reminiscence therapy (RT) intervention adapted from a bereavement-focused platform that uses prompts organized in digital interactive rooms to support dyadic engagement in reflection, storytelling, and connection. The LMH-4-DCP intervention provides caregivers with structured yet open opportunities to reconnect with their care partner, discussing and exploring meaningful moments from their own life and their care partner’s life. This allows for increased connection and helps caregivers remember and feel connected to their care partner’s legacy. In this pilot randomized clinical trial, caregivers were randomized to receive 2-week access to the LMH-4-DCP intervention or an attention control group. Caregivers in the LMH-4-DCP group found the website to be user friendly, with more than 70% of caregivers reporting confidence in using the online platform and moderate-to-strong acceptability. Most caregivers logged onto the intervention platform at least once, although authors did not assess the exact frequency of use. In light of the small analytic sample (n = 54), the authors found preliminary support for LMH-4-DCP in reducing predeath grief between baseline and 2-week follow-up, as well as improved relationship quality that did not achieve clinical significance.

This study extends previous work that has focused primarily on using RT in long-term care settings. RT builds on the assumption that remote memory remains largely intact during the early phases of cognitive decline in individuals with ADRD. This means that individuals with early ADRD may connect with family and friends over old photographs, favorite music, and talk about nostalgic or important moments from their past. For their caregivers, RT facilitates connection to aspects of their care partner’s personality or their relationship that may dissipate as ADRD progresses. In this study, the authors leveraged the RT framework to build on 2 theories: the microsociological theory of adjustment (constructed by several study authors)^4^ and the model of communal coping.^5^ Taken together, these theories suggest that it is crucial to address caregivers’ existential distress related to ADRD losses, which can be facilitated through shared activities that promote social connection. These theories put into context the prebereavement losses experienced by caregivers that hinder social and emotional connection, driving caregivers’ negative mental health outcomes.

Adding the LMH-4-DCP intervention to our therapeutic toolbox would be a welcome addition. Few other interventions exist that target predeath grief in ADRD caregivers; interventions have primarily focused on the bereavement period. However, another intervention, predeath grief therapy, found initial evidence for improved predeath grief and preparedness for patient death, reducing the caregiver’s risk for complicated grief.^6^ Other interventions have been tested in populations with serious illness, such as dignity therapy, meaning-centered psychotherapy, and family-focused support conversation, and target similar constructs of meaning and purpose through discussions of caregiver role, identity, and past experiences.^7^ These interventions are often resource intensive, delivering targeted psychotherapeutic coping skills to confront loss and enhance meaning making through facilitator-led interventions.^6^ In addition, these interventions are time consuming for caregivers—often several 1-h/wk sessions—who are already adding between 10 and 40 hours of caregiving to a full life. These interventions also require significant time for researchers who lead supervision, ensure adherence and intervention quality, and review audiotapes for treatment fidelity. Given the resources of facilitated psychotherapy interventions, difficulty translating research findings to widespread clinical practice, and the national lack of mental health professionals to serve the breadth and depth of care needs in ADRD caregivers, the LMH-4-DCP intervention is a potentially sustainable and accessible intervention for family caregivers outside clinical settings.

While many psychotherapy interventions are now being delivered over technology platforms to provide caregiver-focused psychoeducation and psychotherapeutic support,^8^ digital interventions that use web-based interactive platforms or digital phone applications are slowly but readily increasing access to care. Particularly for the ADRD caregiver population, who are often older adult spousal caregivers, ensuring the usability and age friendliness of such a platform is vital to ensure that these interventions meet all needs of caregivers without adding extra burden. Caregivers in the study by Falzarano et al^3^ had a mean age of 49.4 years, and 39 of the 68 enrolled participants (57.4%) were adult children of the individual with ADRD, suggesting that they may have been more comfortable with using technology than older, spousal caregivers. Participants in this study found the online website to be user friendly and reported confidence in their ability to navigate the website on their own; however, given the relatively younger age of caregivers, it is not clear whether older, spousal caregivers may report the same comfortability with technology.

The LMH-4-DCP intervention and similar web-based interactive interventions hold promise, as they are generally accessible to caregivers, can be used in their own time, and can be revisited throughout the caregiving trajectory. Falzarano et al^3^ do not comment on the usability of the intervention for older (often spousal) caregivers who may experience difficulty or be uncomfortable navigating the webspace. Even more, the experience of predeath grief may differ between adult children and spousal caregivers whose relationship changes differ and whose care responsibilities and interactions may vary in intensity and frequency. Approximately 15% of participants did not log into the digital intervention, suggesting that either caregiving responsibilities impeded participation or caregivers’ perception of potential intervention benefits were not strong enough to lead to initial use. Given the homogenous sample, the applicability of the study findings to more rural areas or racial and ethnic groups is prudent, as these populations often have the greatest difficulty connecting to mental health resources, have the highest level of caregiver burden and distress, and serve to benefit most from this flexible, potent intervention.

Taken together, the LMH-4-DCP intervention is a novel tool that may have powerful effects on improving caregiver mental health, potentially extending to better adjustment in bereavement. Future research is needed to determine who may benefit from these types of digital interventions and which types of caregivers may benefit the most from predeath grief interventions.
